# Risk Factors for Glucose 6-Phosphate Dehydrogenase and COVID-19 Disease—A Retrospective Study at a Major Saudi Tertiary Center

**DOI:** 10.3390/v15061224

**Published:** 2023-05-23

**Authors:** Badi A. Alotaibi, Jehad A. Aldali, Hamzah J. Aldali, Glowi A. Alasiri, Emadeldin M. Elsokkary, Areej Al Mugairi, Abdulaziz M. Almuqrin

**Affiliations:** 1 Department of Clinical Laboratory Sciences, Collage of Applied Medical Sciences, King Saud Bin Abdulaziz University for Health Sciences, Riyadh 11481, Saudi Arabia; 2 King Abdullah International Medical Research Center, P.O. Box 3660, Riyadh 11481, Saudi Arabia; 3 Department of Pathology, College of Medicine, Imam Mohammad Ibn Saud Islamic University (IMSIU), Riyadh 13317, Saudi Arabia; 4 Cellular and Molecular Medicine, College of Biomedical Science, University of Bristol, Bristol BS8 1QU, UK; 5 Department of Biochemistry, College of Medicine, Imam Mohammad Ibn Saud Islamic University (IMSIU), Riyadh 13317, Saudi Arabia; 6 Psychology, Organisation, Imam Mohammed Ibn Saud Islamic University (IMSIU), Riyadh 13317, Saudi Arabia; 7 Hematopathology Division, Department of Pathology and Laboratory Medicine, King Abdulaziz Medical City, Ministry of National Guard Health Affairs, Riyadh 11426, Saudi Arabia; moghairia@ngha.med.sa; 8 Department of Clinical Laboratory Sciences, Collage of Applied Medical Sciences, King Saud University, Riyadh 11433, Saudi Arabia

**Keywords:** glucose-6-phosphate dehydrogenase (G6PD), COVID-19, hematological, biochemical parameters, oxidative stress metabolism

## Abstract

Glucose-6-phosphate dehydrogenase (G6PD) insufficiency is a common enzymatic defect worldwide; it affects over 400 million people and is associated with various disorders. Recent research suggests that G6PD-deficient cells are susceptible to infection by human coronaviruses, as the G6PD enzyme is involved in the metabolism of oxidative stress, which may enhance COVID-19 mortality. This retrospective study aimed to examine the effect of COVID-19 on patients with G6PD deficiency by comparing the laboratory parameters of patients with G6PD enzyme deficiency alone, COVID-19 alone, and those with both COVID-19 and G6PD enzyme deficiency treated at a major Saudi tertiary center. The results indicated significant differences in hematological and biochemical parameters between the three patient groups, indicating that COVID-19 may influence these parameters, and that they could be used to measure the severity of COVID-19 disease. Moreover, this study suggests that patients with G6PD enzyme deficiency may be at higher risk for severe COVID-19 outcomes. Although the study is limited by the lack of a random selection method for group membership, the Kruskal–Wallis H-test was used to statistical assess the data. The study’s findings can enhance the understanding of the relation between COVID-19 infected and G6PD-deficiency patients and inform clinical decision making for an improved patient outcome.

## 1. Introduction

The hexose monophosphate (HMP) shunt, also known as the pentose phosphate pathway (PPP) is an alternative pathway for glucose metabolism. ATP is not produced or consumed directly, but there are two fundamental functions of HMP. The first is the synthesis of ribose 5-phosphate for nucleotide and nucleic acid formation [1,2]. The second is the main source of nicotinamide adenine dinucleotide phosphate (NADPH), a biochemical reductant [2] for synthesis of fatty acids and steroids, and which maintains antioxidant activity through the reduction of glutathione. In addition, glucose-6 phosphate dehydrogenase (G6PD) is an important NADP-dependent enzyme of HMP shunt and catalyzes the rate-limiting step [1]. Deficiency of G6PD is an inherited genetic disorder, X-linked and recessive, in which a G6PD gene mutation leads to inefficient or absent expression and a corresponding deficiency of the enzyme [3]. A study showed that G6PD enzyme deficiency was evidently connected with systolic blood pressure [4]. Moreover, G6PD-deficient patients have 39.6% higher risk of developing cardiovascular disease (CVD) [5,6]. In addition, there are common disorders associated with G6PD enzyme deficiency including acute hemolytic anemia (AHA) (favism), neonatal hyperbilirubinemia (neonatal jaundice), and chronic hemolytic anemia [3,7,8]. G6PD- enzyme deficient patients are usually asymptomatic. However, there are triggers that can potentially cause acute hemolytic anemia. These triggers could be summarized into three: dietary, drug-induced, and, most importantly, pathogenic infections [9]. Dietary triggers are the most common cause of hemolysis in G6PD-deficient individuals; it is mostly caused by fava beans [10]. Second are drug induced triggers; drug-induced hemolytic episodes are somewhat rare compared to dietary triggers. However, they can be lethal to G6PD- enzyme deficient patients [11]. Lastly, are viral infections that produce reactive oxygen species (ROS) through strong inflammation, to which deficient cells are particularly susceptible. At the end of 2019, Severe Acute Respiratory Syndrome Coronavirus 2 (SARS-CoV-2) emerged; this causes Coronavirus Disease 2019 (COVID-19) [12] and appears to cause mild symptoms in the majority of infected people [13]. Nevertheless, the disease frequently develops to severe pneumonia and acute respiratory distress syndrome (ARDS), leading to significant morbidity and mortality [14,15,16]. The infection of human type II alveolar epithelial cells (pneumocytes) is the beginning of the pathophysiological processes of COVID-19. The innate immune response to type II alveolar epithelial cell infection is conducive both to the death of apoptosis and pyroptosis and to the activation of alveolar macrophages. Proinflammatory cytokines and chemokines are secreted by activated macrophages and tend to polarize through the inflammatory M1 phenotype. These alterations are associated with vascular endothelial cell activation and thus the recruitment into the alveolar space of highly toxic neutrophils and inflammatory activated platelets. Activated vascular endothelial cells are a source of proinflammatory cytokines and ROS and contribute to coagulopathy, systemic sepsis, cytokine storm and ARDS development. Moreover, a crucial source of proinflammatory cytokines and ROS is also pulmonary activated platelets [17]. In addition, ROS make a significant contribution to the oxidative damage that exists at the chronic phase of infection and is involved in functional impairment of the different tissues. These ROS often alternate normal biological roles of biological molecules. In order to suppress ROS and minimize oxidative damage, antioxidant enzyme mechanisms have developed. Defects in essential antioxidant enzymes such as G6PD, whether inherited or acquired, trigger a dysregulated redox environment, which promotes pathobiology [18].

Excluding COVID-19 deaths in the elderly and chronically ill, 0.9% of COVID-19 deaths had no identified chronic disease [19]. One of the health conditions that may increase the risk of death in people infected with COVID-19 is glucose-6-phosphate dehydrogenase (G6PD) enzyme deficiency, the most common enzyme deficiency in the world. It affects more than 400 million people and causes various diseases [20]. Wu et al. reported that G6PD-deficient cells were infected with human coronavirus (HCoV) 229E at a higher rate compared to normal cells [21]. The putative role of G6PD in oxidative stress metabolism may explain the influence of G6PD enzyme deficiency on viral illnesses. G6PD is the rate-limiting enzyme of the pentose phosphate pathway and is involved in the reduced glutathione (GSH)/oxidized glutathione (GSSG) balance in both the cytosol and mitochondria to generate nicotinamide adenine dinucleotide phosphate (NADPH). Glutathione metabolism is a key component of the human antioxidant defense system. Thus, reduced G6PD levels lead to increased oxidative stress and impair the redox imbalance [22].

Previous studies have confirmed that viral infection induces the production of reactive oxygen species (ROS) and reactive nitrogen species (RNS), both of which, when the metabolism of antioxidant enzymes is compromised, reduce cellular protein production and damage their host’s DNA and cellular components. Because G6PD enzyme deficiency causes a redox imbalance in red blood cells, leading to hemolysis and tissue damage due to insufficient oxygen transport, COVID-19 may increase the risk of mortality in patients with G6PD enzyme deficiency [20,21,22]. The severity of COVID-19 is influenced by genetic variants in human G6PD, which are associated with impaired immune responses [23]. It was predicted that COVID-19 would spread more widely in regions or countries with high incidence rates of G6PD enzyme deficiency, making the treatment and control of the COVID-19 outbreak more challenging. Saudi Arabia is one of the countries that have high incident rates of G6PD deficiency among the population [24]. Severe G6PD enzyme deficiency is associated with altered immune responses, including neutrophil extracellular traps formation and inflammasome activation, which present a challenge during the COVID-19 pandemic [24]. However, the association between G6PD enzyme deficiency and the severity of COVID-19 infection remains to be elucidated [19]. This study aims to explore the relationship between G6PD enzyme deficiency and COVID-19 infection in reference to hematological and biochemical parameters among patients managed at a Saudi tertiary center.

## 2. Method

This is a retrospective study involving individuals who tested positive for COVID-19 using polymerase chain reaction tests, G6PD enzyme deficiency patients, or G6PD enzyme deficiency patients who also tested positive for COVID-19 from the period of April 2020 to February 2022 in the ministry of National Guard—Health affairs, Saudi Arabia. The clinical data such as prothrombin time/international normalized ratio PT/INR, partial thromboplastin time (PTT), D-dimer, fibrinogen, total bilirubin, aspartate aminotransferase (AST), alanine aminotransferase (ALT), albumin, creatinine, blood urea nitrogen (BUN), glucose, and C-reactive protein (CRP) were included. The data were retrieved from the department of research data management section in the King Abdullah International Medical Research Center (KAIMRC) in the ministry of National Guard—Health affairs, Riyadh, Saudi Arabia. The inclusion criteria were a diagnosis of G6PD enzyme deficiency and COVID-19 patient’s, with G6PD enzyme deficiency patients in Saudi and non-Saudi patients and all age groups. All records that did not include any of the above variables were excluded from the study. The ethical approval for the study was obtained from the institutional review board at KAIMRC under the approval number (IRB/0391/22).

## 3. Results 

### Comparison between COVID-19 Patients with and without G6PD 

The study analyzed three groups of patients who were available in the institution (Table 1). The first group represents a total number of 42,856 COVID-19 patients. The second group represents 233 G6PD deficiency patients, while the third group represents 21 patients with G6PD deficiency and COVID-19. The proportion of female patients is slightly higher than male patients in the COVID-19 group. On the other hand, the proportions of male patients in the G6PD deficiency group and in the G6PD deficiency with COVID-19 group were significantly higher than the female patients (75.5%) and (66.6%), respectively.

The distribution of age groups in the G6PD deficiency group was as follows: 57.5% of patients were below 15 years of age, 15.02% of patients were between 15 and 30 years of age, and 13.7% of patients were between 30 and 50 years of age. The remaining 9.4% of patients were between 50 and 80 years of age.

In the group of patients with G6PD deficiency and COVID-19, 52.38% of patients were below 15 years of age, 14.28% were between 15 and 30 years of age, and 19.04% of patients were between 30 and 50 years of age. The remaining (14.28%) patients were between 50 and 80 years of age. In the COVID-19 patients’ group without G6PD deficiency, 12.97% of patients were below 15 years of age, 26.66% were between 15 and 30 years of age, 36.18% of patients were between 30 and 50 years of age, and 25.34% of patients were between 50 and 80 years of age. Interestingly, a tiny proportion of patients (0.095%) were above 80 years.

In the group of 233 G6PD deficiency patients, several laboratory parameters including PT (50 patients, 21.46%), PTT (46 patients, 19.64%), D-dimer (20 patients, 8.85%), fibrinogen (12 patients, 5.15%), lactate dehydrogenase (LDH) (74 patients, 1.76%), total bilirubin (141 patients, 60.52%), ferritin (95 patients, 40.77%), and aspartate aminotransferase (AST) (138 patients, 95.2%) were evaluated. In the second group of 21 COVID-19 patients with G6PD, tests were conducted on PT (47.62%), PTT (33.33%), fibrinogen (19.05%), total bilirubin (76.19%), ferritin (76.19%), aspartate aminotransferase (76.19%), and alanine aminotransferase (66.67%). In the third group of 42,856 G6PD patients, tests for PT were conducted on 8375 people (19.54%), PTT on 191 people (0.45%), D-dimer on 20,815 people (48.57%), fibrinogen on 1585 people (3.70%), LDH on 826 people (1.93%), blood glucose on 278 people (0.65%), and creatinine on 1955 people (4.56%) (see Table 1 and Table 2).

For PT, the mean is higher in G6PD group compared to a G6PD with COVID-19 and COVID-19 groups (11.55 vs. 11.43 vs. 11.47). However, the standard deviation is increased in the COVID-19 patient group (3.56) compared to other groups. For PTT, the mean and standard deviation are higher in the COVID-19 patients’ group compared to others (28.31 and 4.55), which is similar to fibrinogen (4.49 and 1.98). In D-dimer, the mean is higher in G6PD patients with COVID-19 (2.95) compared to other groups. However, standard deviation is increased in the COVID-19 patients’ group (3.66) compared to other groups. For LDH, the mean and standard deviation are higher compared to other groups (614.77 and 516.32). For total bilirubin, the mean and standard deviation are higher in G6PD group (27.51 and 36.26) compared to other groups. For ferritin, the mean and standard deviation are higher in G6PD group with COVID-19 (1977 and 4750.85) and similarly for AST (39.56 and 34.75). For ALT, the mean is higher G6PD with COVID-19 (34.39) compared to other groups, while the standard deviation is increased in the G6PD group (44.28) compared to others. For albumin, the mean is increased in the G6PD group (42.43) compared to other groups, whereas the standard deviation is increased in G6PD with COVID-19 (6.28) compared to others. For creatinine, the mean and standard deviation are higher in COVID-19 patients (125.61 and 175.73) compared to others. For blood urea nitrogen, the mean and standard deviation are higher in the G6PD patients with COVID-19 group (6.17 and 8.65) compared to others. For glucose, the mean and standard deviation are higher in COVID-19 patients (6.16 and 2.78) compared to others. Similarly for CRP (50.39 and 78.75) see Table 3, the results of the study indicate some significant differences in the mean and standard deviation of various blood tests among the three groups of patients. Researchers used the Kruskal–Wallis H-test as an alternative to a one-way analysis of variance (one-way ANOVA) because the number of measurements in the G6PD + COVID-19 group ranged from 4 to 18 measurements and due to the existence of a considerable disparity in numbers between the three research groups, as well as the absence of a random selection process for group membership.

Table 4 shows multiple-dimensional comparisons between the mean ranks of each of the two groups of the three groups separately in each of the measurements, and the following is evident from it: For PT-, there are significant differences between the (G6PD) and (COVID-19) groups in the direction of the (G6PD) group, and there are no significant differences in the rest of the bilateral comparisons. For D-dimer-, there are significant differences between the (G6PD) and (COVID-19) groups in the direction of the (G6PD) group, and there are no significant differences in the rest of the binary comparisons. For fibrinogen-, there are significant differences between the (G6PD) and (COVID-19) groups towards the (COVID-19) group, and there are no significant differences in the rest of the bilateral comparisons. Regarding LHD, there are significant differences between the (G6PD + COVID-19) and (COVID-19) groups in the direction of the (G6PD + COVID-19) group, and there are no significant differences in the rest of the bilateral comparisons. For Tbili-, there are significant differences between (G6PD) and (COVID-19) groups in the direction of (G6PD) group, there are significant differences between (G6PD + COVID-19) and (COVID-19) groups in the direction of (G6PD + COVID-19) group, and there are no significant differences between the two groups (G6PD), (G6PD + COVID-19).

For ferritin-, there are significant differences between the (G6PD) and (COVID-19) groups in the direction of the (COVID-19) group, and there are significant differences between the two groups (G6PD), (G6PD + COVID-19), in the direction of the (G6PD) group. There are no significant differences between the two groups (COVID-19) and (G6PD + COVID-19). For AST-, there are significant differences between the (G6PD) and (COVID-19) groups towards the (G6PD) group, and there are significant differences between the (G6PD + COVID-19) and (COVID-19) groups towards the (G6PD + COVID-19) group, but there are no significant differences between the two groups (G6PD), (G6PD + COVID-19). Regarding ALT-, there are significant differences between the (G6PD) and (COVID-19) groups in the direction of the (COVID-19) group, and there are no significant differences in the rest of the bilateral comparisons. As for creatinine, there are significant differences between the (G6PD) and (COVID-19) groups in the direction of the (COVID-19) group, there are significant differences between the (G6PD + COVID-19) and (COVID-19) groups in the direction of the (COVID-19) group, but there are no significant differences between the two groups (G6PD), (G6PD + COVID-19). For BUN- there are significant differences between the (G6PD) and (COVID-19) groups in the direction of the (COVID-19) group and there are no significant differences in the rest of the bilateral comparisons. These results indicate that COVID-19 might influence the hematological and biochemical parameters, which could be used to observe the severity of COVID-19 disease.

## 4. Discussion

The role of G6PD enzyme deficiency in viral infections has been extensively studied [20,21,22,25]. NADPH cofactor plays an important role in suppressing free radicals by balancing glutathione (GSH) and oxidized glutathione (GSSG). This takes place in both the cell’s cytoplasm and mitochondria. Decreased G6PD levels lead to decreased NADPH levels and increased free radical levels due to inadequate neutralization processes by GSH. Clinically, oxidative stress manifests itself in hemolysis and its complications. There has been little prior literature conducted evaluating the effect of COVID-19 on G6PD deficiency patients. A recent study examined the effect of COVID-19 on a group of G6PD patients, based only on the hematological parameters [26]. Another study conducted in 2021 evaluated the difference in hematological and some biochemical parameters between only two COVID-19 cases, with or without G6PD deficiency underlying condition [27]. Compared with the previously mentioned studies, our study provides a comparative observational view regarding the effects of COVID-19 on G6PD deficiency patients not only based on hematological parameters but also based on biochemical parameters, with a larger number of patients. Moreover, this study is the first study evaluating the effect of COVID-19 on Saudi and non-Saudi G6PD deficiency patients at a major Saudi tertiary center. Our study suggested that both biochemical and hematological data can be used to predict the severity of COVID-19 among G6PD deficiency patients.

In this study, we investigated the relationship between COVID-19 and glucose-6-phosphate dehydrogenase (G6PD) deficiency by analyzing the different hematological and biochemical parameters of 233 G6PD deficiency patients, 21 COVID-19 patients with G6PD deficiency, and 42,856 COVID-19 patients.

Our study’s findings on the higher proportion of males in the G6PD deficiency patients’ group and in the G6PD with COVID-19 patients’ group are consistent with previous research showing a higher prevalence of G6PD deficiency among males than females [28,29]. This observation suggests that males with G6PD deficiency may be more likely to contract COVID-19 than G6PD deficiency female patients. However, the higher proportion of females in the COVID-19 group may reflect a greater susceptibility of females to respiratory infections than males in non-G6PD individuals, or differences in social and environmental factors that may impact COVID-19 transmission in females in Saudi Arabia. In addition, this study showed that most G6PD patients with COVID-19 were below 15 years of age and a very low proportion of G6PD individuals with COVID-19 were above 80 years of age. One possible explanation for the last observation is that G6PD-deficient individuals above 80 years of age may have a higher risk of mortality from COVID-19 and, therefore, may not have been included in the study sample. However, this observation needs further investigation.

The results revealed significant differences in several hematological and biochemical parameters between the three groups. The mean and standard deviations of laboratory parameters, such as PT, fibrinogen, D-dimer, LDH, total bilirubin, ferritin, AST, ALT, albumin, creatinine, and blood urea nitrogen were calculated. The level of these hematological and biochemical parameters was different in the three groups, and COVID-19 might influence the difference in these parameters. These findings suggest that these laboratory parameters could be used to observe the severity of COVID-19 disease in patients with G6PD enzyme deficiency. This might be attributed to the role that oxidative stress-related problems played in coronavirus-induced cell death [21].

An in vitro study published in 2008 examined the effects of the human coronavirus HCoV229E on G6PD-deficient cells. In that study, the researchers revealed that infected HCoV229E virus cells that lack G6PD production had increased oxidant production, indicating cellular stress, and the infected cells lacking G6PD had a higher viral replication rate [21]. It is important to note that COVID-19 causes cell death by triggering a pro-inflammatory systemic response. Inflammation is known to cause oxidative stress [30]. This results in excessive production of oxygen free radicals. These radicals further stimulate the systemic inflammatory response syndrome. A cross-sectional analysis examining various laboratory parameters in COVID-19 cases was performed [31,32]. In this study, it was found that the increase of CRP, LDH levels and the drop of albumin level in the blood are laboratory parameters that were associated with increased COVID-19 severity. This might indicate a possible worse outcome for patients with G6PD enzyme deficiency.

In this study, we showed that the three studied patient groups differed significantly in the number of haematological and biochemical parameters. The three groups had varied mean and standard deviation values for laboratory tests such PT, fibrinogen, D-dimer, LDH, total bilirubin, ferritin, AST, ALT, albumin, creatinine, and blood urea nitrogen, suggesting that COVID-19 may have had an impact on these measurements. According to these results, these laboratory measurements could be utilized to monitor the severity of COVID-19 disease in G6PD individuals. In addition, the large cohort of group of COVID-19 patients without G6PD condition will provide valuable support to the findings of the study groups.

However, there are a few limitations in this study: First, the number of cases of G6PD deficiency with COVID-19 group and G6PD deficiency group are relatively small, which may affect the generalizability of the findings. Second, the study only included G6PD patients, limiting the generalizability of the findings to the broader population. Third, correlation does not imply causation. However, a positive correlation indicates a subset of patients who are susceptible or at high risk of contracting COVID-19 [26,33]. Future studies need to include a larger sample size, a broader patient population, and a more controlled study design to confirm these findings. Despite the mentioned limitations, our study provides preliminary evidence of the relationship between G6PD and a higher susceptibility of contracting COVID-19 and may guide future research on the impact of COVID-19 on hematological and biochemical parameters. Further research in this area is warranted to validate these findings and establish strategies for early identification and management of patients with G6PD enzyme deficiency and COVID-19.

## 5. Conclusions

Current research indicates that G6PD-deficient cells are more susceptible to human coronavirus infection than normal cells due to G6PD’s role in the metabolism of oxidative stress, which could increase COVID-19 mortality risk. Our results demonstrated substantial changes in haematological and biochemical parameters across the three studied patient groups, indicating that COVID-19 may alter these indicators, which might be useful in determining the severity of COVID-19. In addition, this study implies that individuals with G6PD deficiency may be at an increased risk for COVID-19 contraction and complications. Despite the absence of a random selection technique for group membership, the Kruskal–Wallis H-test was utilized to analyze the data. Additional study is required to validate our findings and to establish strategies for early detection and management of patients with G6PD and COVID-19. The results of this study may contribute to a greater comprehension of the COVID–G6PD association and inform therapeutic decision-making to improve patient outcomes.

## Figures and Tables

**Table 1:** Demographic characteristics of study participants.

Gender/Age	G6PD Group (233) Number/Percentage	G6PD Group + COVID-19 [21] Number/Percentage	COVID-19 (42,856) Number/Percentage
Male	176/75.5%	14/66.6	20,647/48.17
Female	57/24.4	7/33.33	22,206/51.81
UNKNWON	NA	NA	2/0.004
<15	134/57.5	11/52.38	5559/12.97
>15 <30	35/15.02	3/14.28	11,413/26.663
>30 <50	32/13.7	4/19.04	15,507/36.18
>50 <80	22/9.4	3/14.28	10,860/25.34
>80	NA	NA	41/0.095

**Table 2:** Numbers and percentages of each measurement in relation to the total number of people in each of the three groups: COVID-19, G6PD group, and G6PD with COVID-19.

	G6PD Group (233) Number/Percentage	G6PD Group + COVID-19 [21] Number/Percentage	COVID-19 (42,856) Number/Percentage
PT	50/21.46	11/52.38	8375/19.54
PTT	46/19.64	10/47.62	191/0.45
D-DIMER	20/8.58	7/33.33	20,815/48.57
Fibrinogen	12/5.15	4/19.05	1585/3.70
LHD	74/31.76	13/61.90	826/1.93
Total bilirubin	141/60.52	16/76.19	290/0.68
Ferritin	95/40.77	11/52.38	921/2.15
AST	138/59.23	16/76.19	524/1.22
ALT	112/48.07	14/66.67	363/0.85
Creatinine	162/69.53	18/85.71	1955/4.56
Blood Urea Nitrogen (BUN)	159/68.24	18/85.71	204/0.48
Blood Glucose Test	151/64.81	17/80.95	278/0.65
C-Reactive Protein (CRP)	68/29.18	10/47.62	807/1.8

**Table 3:** Arithmetic means and standard deviations for each of the measurements obtained for each of the three groups. G6PD group, G6PD group + COVID-19 [21], and COVID-19 group.

	G6PD		G6PD + COVID-19		COVID-19	
	Mean	Std. Deviation	Mean	Std. Deviation	Mean	Std. Deviation
PT second	11.55	1.25	11.43	1.31	11.47	3.56
PTT second	27.73	3.26	26.35	2.08	28.31	4.55
D-DIMER mg/L	2.07	2.46	2.95	3.52	1.92	3.66
Fibrinogen gm/L	3.26	1.39	3.33	1.92	4.49	1.98
LHD U/L	466.07	462.34	614.77	516.32	321.70	221.56
Tbili μmol/L	27.51	36.26	21.94	23.63	9.66	5.34
Feeritin ug/L	565.91	1811.98	1977.11	4570.85	545.53	1660.98
AST U/L	32.56	36.23	39.56	34.75	31.29	37.12
ALT U/L	29.69	44.28	34.21	34.39	28.99	40.01
Albumin g/L	42.43	4.60	37.85	6.28	40.68	5.10
Creatinin μmol/L	63.94	60.16	74.67	69.34	125.61	175.73
BUN mmol/L	4.27	3.24	6.17	8.65	5.71	4.01
GLU Random	5.46	2.49	6.08	2.63	6.16	2.78
CRP mg/L	35.71	53.97	25.32	50.47	50.39	78.75

**Table 4:** All pairwise multiple comparisons of groups: G6PD group, G6PD Group + COVID-19 group, and COVID-19 group.

	Group	G6PD		G6PD + COVID-19		COVID-19	
		Std. Test Statistic	*p* Value	Std. Test Statistic	*p* Value	Std. Test Statistic	*p* Value
PT	G6PD	-	-	0.644	0.519	2.736	0.006
	G6PD + COVID-19			-	-	0.575	0.565
	COVID-19					-	-
D-DIMER	G6PD	-	-	−0.220	0.826	2.029	0.042
	G6PD + COVID-19			-	-	1.457	0.145
	COVID-19					-	-
Fibrinogen	G6PD	-	-	−0.086	0.932	−2.218	0.027
	G6PD + COVID-19			-	-	−1.185	0.236
	COVID-19					-	-
LHD	G6PD	-	-	−1.890	0.059	1.190	0.234
	G6PD + COVID-19			-	-	2.550	0.011
	COVID-19					-	-
Tbili	G6PD	-	-	0.515	0.607	6.670	<0.001
	G6PD + COVID-19			-	-	2.138	0.033
	COVID-19					-	-
Feeritin	G6PD	-	-	−2.490	0.013	−2.483	0.013
	G6PD + COVID-19			-	-	1.733	0.083
	COVID-19					-	-
AST	G6PD	-	-	−1.421	0.155	2.599	0.009
	G6PD + COVID-19			-	-	2.458	0.014
	COVID-19					-	-
ALT	G6PD	-	-	−1.540	0.124	−2.533	0.011
	G6PD + COVID-19			-	-	0.597	0.550
	COVID-19					-	-
Creatinin	G6PD	-	-	−0.324	0.746	−8.657	0.000
	G6PD + COVID-19			-	-	−2.649	0.008
	COVID-19					-	-
BUN	G6PD	-	-	−0.803	0.422	−4.317	<0.001
	G6PD + COVID-19			-	-	−1.045	0.296
	COVID-19					-	-

## Data Availability

Might be provided upon request to the corresponding author.
